# An effective endoscopic hand-suturing method for fistula closure after endoscopic transgastric drainage for walled-off necrosis

**DOI:** 10.1016/j.vgie.2024.03.003

**Published:** 2024-03-09

**Authors:** Yorihito Hayashi, Hideki Nagumo, Ai Fujimoto, Kensuke Takuma, Takahisa Matsuda

**Affiliations:** 1 Division of Gastroenterology and Hepatology, Toho University Omori Medical Center, Tokyo, Japan

## Abstract

Video 1 How to use an endoscopic hand-suturing device (video version).

How to use an endoscopic hand-suturing device (video version).

## Background

Endoscopic hand-suturing is a novel method developed by Goto et al^1^ to enable endoscopic suturing of a mucosal defect using a curved needle and needle holder. Endoscopic hand-suturing is useful for providing traction when incising gastric submucosal tumors.^2^ Feasibility studies have demonstrated that ulcers that occur after endoscopic submucosal dissection (ESD) could be closed using endoscopic hand-suturing.^3^ Furthermore, endoscopic hand-suturing for ulcers that occur after ESD has been reported to promote healing in the porcine stomach and may prevent postoperative bleeding in patients receiving antithrombotic therapy.^4^^,^^5^ However, closure of fistulas caused by walled-off necrosis attributable to acute pancreatitis by transgastric endoscopic hand-suturing has rarely been reported.^6^

Endoscopic suturing methods include the X-Tack Endoscopic HeliX Tacking System (Apollo Endosurgery, Inc, Austin, Tex, USA), which can close mucosal defects, and Apollo Overstitch SX (Apollo Endosurgery, Inc), which can cover the entire thickness of mucosal defects; however, in this case we used SutuArt (Olympus Corp, Tokyo, Japan) to close the fistula.

Written informed consent was obtained from the patient for publication of this case report and the accompanying images.

## Case Presentation

**Figure 1:**
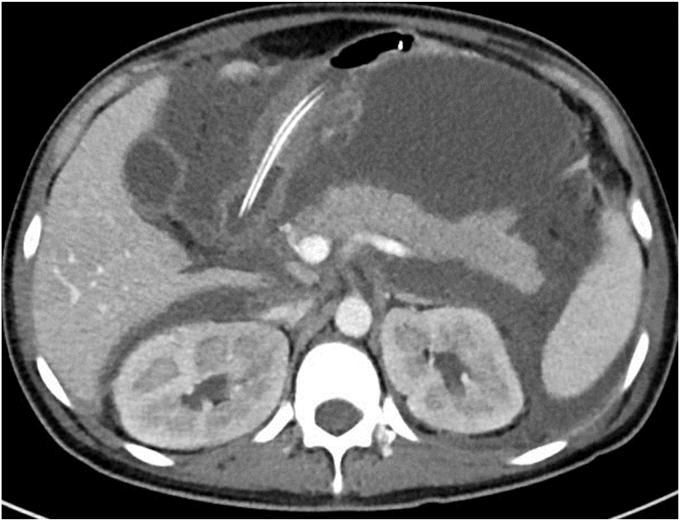
CT image at the time of admission. Inflammatory spillover throughout the abdominal cavity.

**Figure 2:**
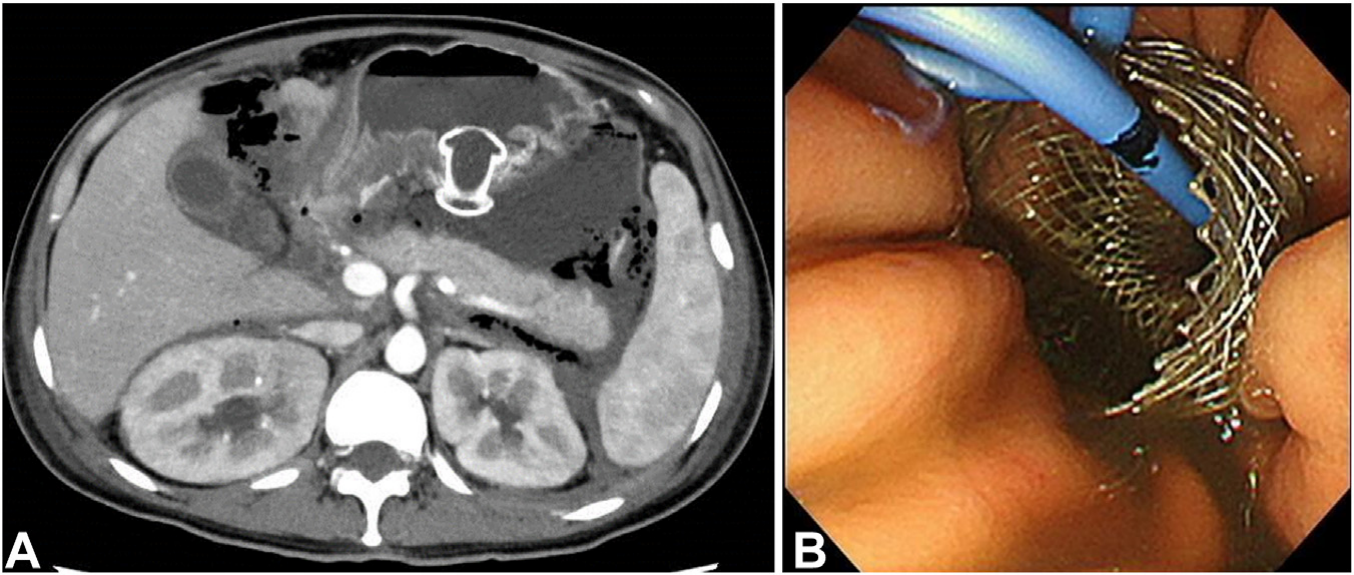
Lumen-apposing metal stent (LAMS) placement. **A,** Placement of a LAMS to allow fluid collection around the pancreas. **B,** Double-pigtail plastic stent placement within the LAMS.

A 35-year-old woman was hospitalized with severe acute pancreatitis associated with hypertriglyceridemia (Fig. 1). On day 20 of hospitalization, she underwent endoscopic necrosectomy using a lumen-apposing metal stent (LAMS) (Hot Axios; Boston Scientific, Boston, Mass, USA) to treat walled-off necrosis (Fig.  2).

On day 48 of hospitalization, the LAMS was removed and 6 Zimmon Pancreatic Stents (Cook Medical, Bloomington, Ind, USA) were placed to prevent fistula closure. Necrosectomy was repeated 9 times in total in the period lasting until day 90. After the removal of the pancreatic stent, oral intake was resumed, and food intake was increased.

Necrosectomy and antimicrobial therapy were then continued, followed by an improvement in inflammatory response. However, the fistula failed to close, and the ingested contents leaked into the abdominal cavity, causing a worsening of the inflammatory reaction.

**Figure 3:**
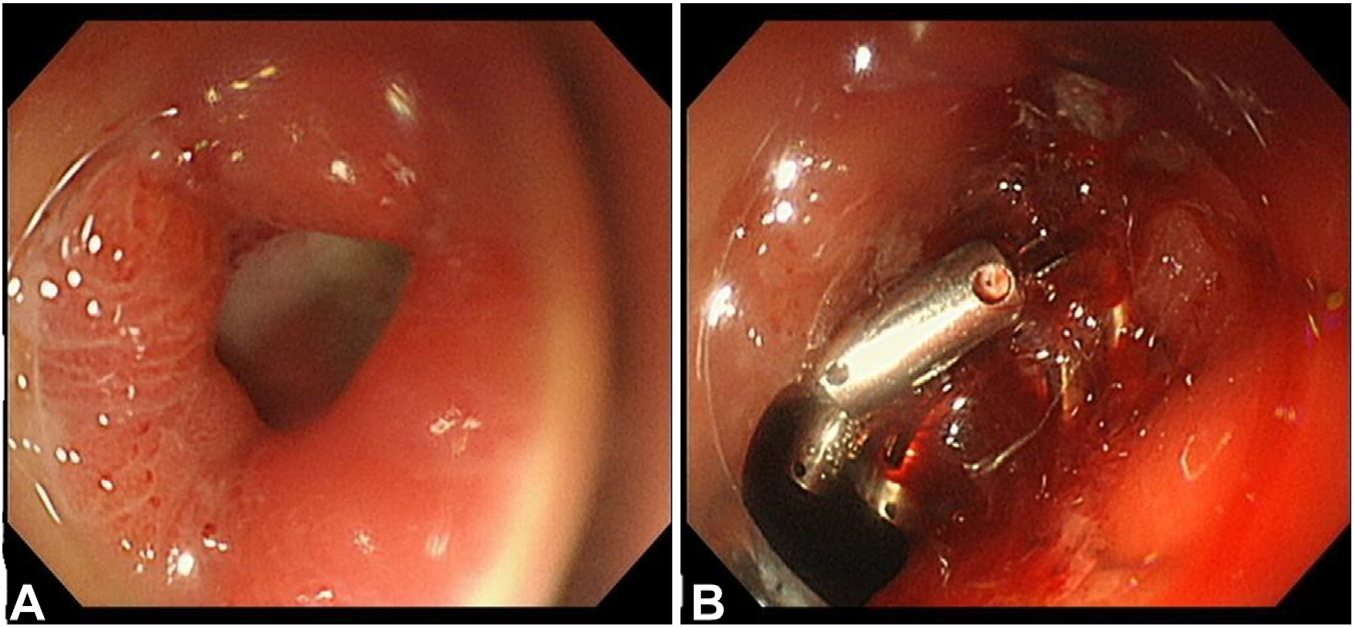
Failure of the fistula to close. **A,** A patent fistula at the lumen-apposing metal stent placement site. **B,** The gastric mucosa around the fistula is hard, and the clip cannot completely close the fistula.

On day 132 of hospitalization, closure of the fistula site with a SureClip (Micro-Tech Corp, Nanjing, China) was attempted. However, the procedure was not successful, as the fistula was surrounded by fibrotic tissue and the clip partially strayed into the abdominal cavity (Fig. 3). On day 171 following admission, fistula closure was attempted using endoscopic hand-suturing.

## Technique

**Figure 4:**
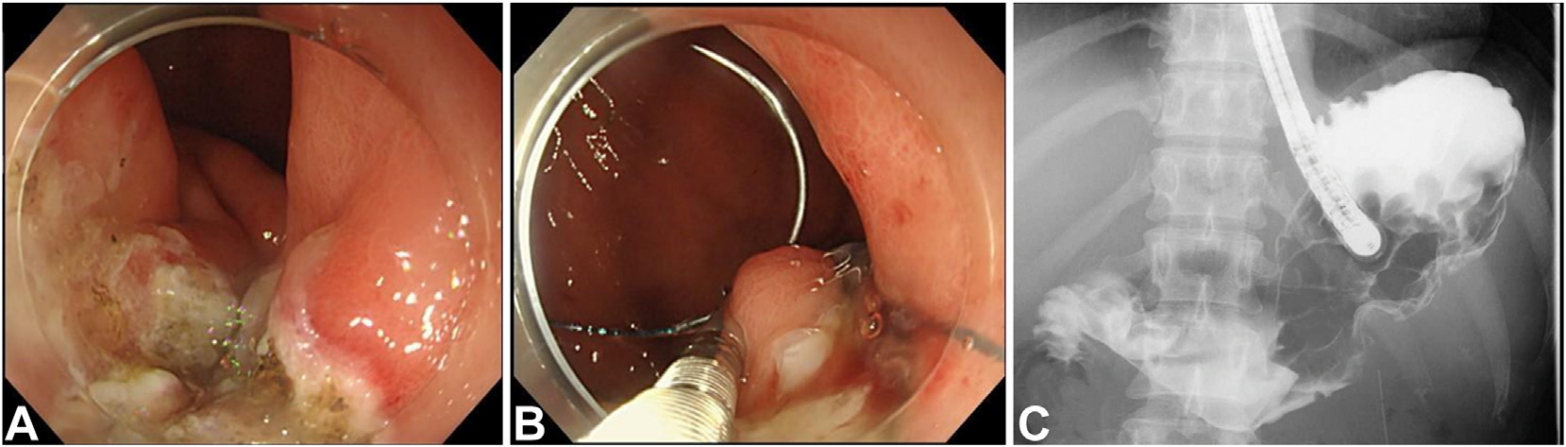
How to use an endoscopic hand-suturing device. **A,** The gastric mucosa around the fistula is cauterized circumferentially using an electrical device. **B,** The fistula is completely sutured. **C,** Fluoroscopy confirms no contrast leakage into the abdominal cavity.

To promote wound healing, the gastric mucosa around the fistula was cauterized with argon plasma coagulation before suturing. Continuous stitching was then performed from the anal side to the oral side of the fistula, and 3 stitches were sewn close to the fistula, with a total operative time of 120 minutes. Finally, Gastrografin (Bracco Diagnostics, Monroe Township, NJ, USA) was sprayed into the stomach to confirm that there was no leakage into the abdominal cavity (Fig. 4; Videos 1 and 2, available online at www.videogie.org).

## Results

**Figure 5:**
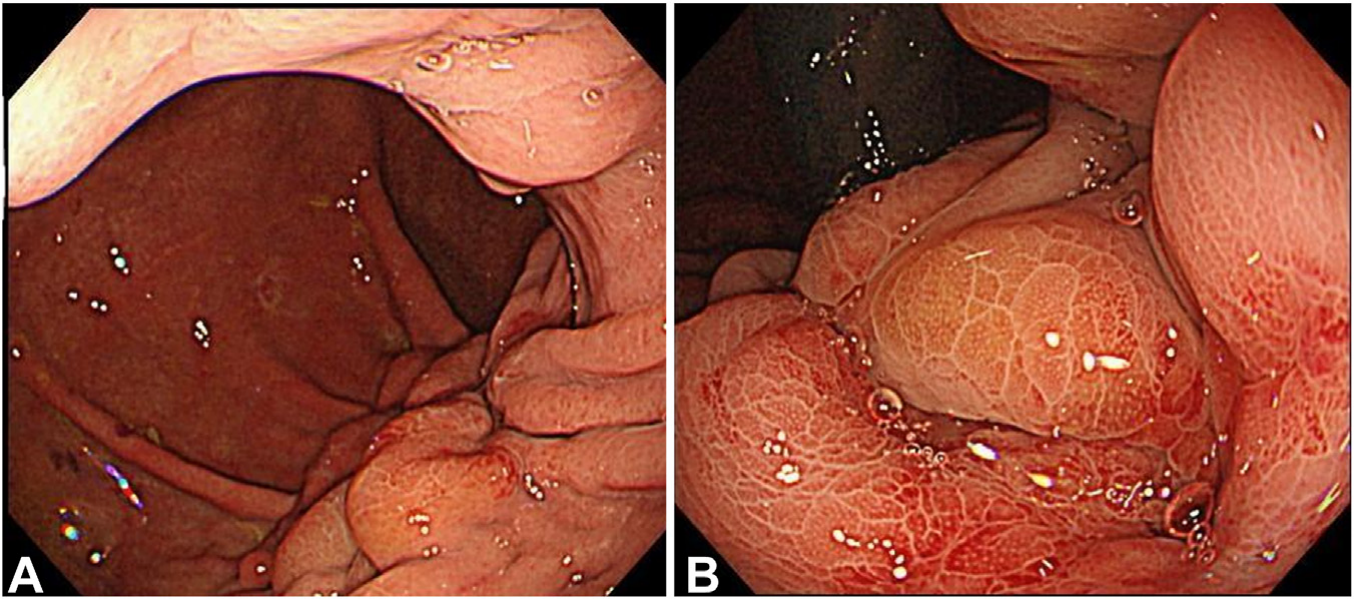
Endoscopic findings at time of discharge. **A,** After endoscopic suturing, the fistula is completely closed. **B,** Close-up first look at the fistula closure site.

After the resumption of oral food intake, a blood examination confirmed that inflammatory reactions remained normal. On day 179 of hospitalization, complete closure of the fistula was confirmed by EGD, and the patient was discharged (Fig. 5).

## Discussion

Using endoscopic hand-suturing, we successfully closed a fistula that was created after the withdrawal of a LAMS. Suturing a fistula using endoscopic hand-suturing is technically more difficult than suturing the submucosal layer of an ulcer after ESD. Although suturing the layers of the stomach using endoscopic hand-suturing is time consuming, we believe that the method of coagulating the fistula margins with argon plasma coagulation followed by suturing the entire layer of the fistula with endoscopic hand-suturing is very useful for ensuring that the suture site will not break apart again.

## Disclosure

The authors disclosed no financial relationships relevant to this publication.

## Supplementary data

Video 1 How to use an endoscopic hand-suturing device (video version).

Video 2 Under fluoroscopy, Gastrografin was sprayed into the stomach.
